# The complete mitochondria genome of *Chrysomya phaonis* (Seguy, 1928) (Diptera: Calliphoridae)

**DOI:** 10.1080/23802359.2016.1233466

**Published:** 2017-01-04

**Authors:** Jian Chen, Deyi Qiu, Qiaoyun Yue, Chuanxian Wang, Xiaohong Li

**Affiliations:** a Zhongshan Entry-Exit Inspection and Quarantine Technology Center, Zhongshan, Guangdong, P.R. China;; b Shanghai Entry-Exit Inspection and Quarantine Bureau, Shanghai, P.R. China

**Keywords:** *Chrysomya phaonis* (Seguy,1928), mitochondria genome, molecular identification

## Abstract

*Chrysomya phaonis* (Seguy, 1928) is one of the blowflies of great medical and forensic importance. In this paper, we report that the entire genome of mitochondrial DNA of *C. phaonis* is 15,831 bp in length, which consists of 39 genes including13 protein-coding genes, 24 tRNA genes, 2 mitochondrial ribosomal RNA genes, and a 992 bp non-coding A + T-rich region. The overall base compositions of A, G, C, and T are 38.79%, 9.75%, 14.15%, and 37.31%, respectively. We provide the first complete mitochondrial genome of *C. phaonis,* and should provide useful information for phylogenetic and species identification for *C. phaonis*.

Blowflies (Diptera: Calliphoridae) are distributed worldwide, some species play important role in the mechanical transfer of disease to human beings and animals (Norris 1965; Zumpt 1965; Greenberg 1971; Greenberg 1973; Kuhlhorn 1983; Ghandour 1988). *Chrysomya phaonis* (Seguy, 1928) is one of the most important oriental species of the blowflies, *C. phaonis* distributes in China, India, Nepal, and Afghanistan (Fan 1992; Fan et al. 1997; Kurahashi et al. 1994). However, there is limited molecular biology information about this species. Viewing it is a potential medical vector and indictor in forensic science, we report here the complete mitochondrial genome of *C. phaonis* for species identification and phylogenetic analysis.

Exampled sample of *C. phaonis* were obtained in Bayi County, Tibet, China (N29°37′31.39″; E94°23′25.01″) in July 2013. The studied specimen is stored in the medical vector collections of Zhongshan Entry-Exit Inspection and Quarantine technology center, and the accession number to the specimen is 20130728-232F-Baiyi. We designed 10 pairs of oligo-nucleotide primers according to the conserved regions from reported mitochondria genome sequences of its most related species *C. pinguis.*

The complete mitochondrial genome of *C. phaonis* (GenBank accession KX500359) is 15,831 bp in length, which consists of 39 genes (Table 1) including 13 protein-coding genes, 24 tRNA genes, 2 mitochondrial ribosomal RNA genes (12S rRNA and 16S rRNA), and a 992 bp non-coding A + T-rich region. The 13 protein-coding genes include seven NADH dehydrogenase (*ND1-6* and *ND4L*), three subunits of cytochrome oxidase (*COI-III*), two subunits of ATP synthase (*ATP4* and *ATP6*), one subunit of cytochrome b (*Cytb*). Twelve of the 13 protein-coding genes were identified with ATN as start codon coding for M except for *COI*, which is similar to the former studies (Yan et al. 2014; Zhong et al. 2016).

**Table 1.** Mitochondrial gene profile of *C. phaonis*.

Gene	Direction	Nucleotidenumber	Size(bp)	OL	Non	Anticodon	Codon Start Stop	
*tRNA^Ile^*	J	1–64	64		4	GAT		
*tRNA^Gln^*	N	69–137	69		8	TTG		
*tRNA^Met^*	J	146–214	69			CAT		
*ND2*	J	215–1229	1015				ATT	T
*tRNA^Trp^*	J	1230–1297	68	8		TCA		
*tRNA^Cys^*	N	1290–1353	64		7	GCA		
*tRNA^Tyr^*	N	1361–1426	66	2		GTA		
*COI*	J	1425–2958	1534				TCG	T
*tRNA^Leu(UUR)^*	J	2959–3024	66		5	TAA		
*COII*	J	3030–3717	688				ATG	T
*tRNA^Lys^*	J	3718–3788	71	1		CTT		
*tRNA^Asp^*	J	3788–3854	67			GTC		
*ATP8*	J	3855–4019	165	7			ATT	TAA
*ATP6*	J	4013–4690	678		4		ATG	TAA
*COIII*	J	4695–5483	789		6		ATG	TAA
*tRNA^Gly^*	J	5490–5554	65			TCC		
*ND3*	J	5555–5908	354		2		ATT	TAA
*tRNA^Ala^*	J	5911–5975	65·	1		TGC		
*tRNA^Arg^*	J	5975–6038	64		6	TCG		
*tRNA^Asn^*	J	6045–6110	66	1		GTT		
*tRNA^Ser(AGN)^*	J	6110–6179	70			GCT		
*tRNA^Glu^*	J	6180–6247	68		18	TTC		
*tRNA^Phe^*	N	6266–6332	67			GAA		
*ND5*	N	6333–8052	1720		15		ATT	T
*tRNA^His^*	N	8068–8133	66			GTG		
*ND4*	N	8134–9472	1339	7			ATG	T
*ND4L*	N	9466–9762	297		2		ATG	TAA
*tRNA^Thr^*	J	9765–9829	65			TGT		
*tRNA^Pro^*	N	9830–9895	66		2	TGG		
*ND6*	J	9898–10,422	525	1			ATT	TAA
*Cytb*	J	10,422–11,556	1135				ATG	T
*tRNA^Ser(UCN)^*	J	11,557–11,624	68		16	TGA		
*ND1*	N	11,641–12,579	939		10		ATA	TAA
*tRNA^Leu(CUN)^*	N	12,590–12,654	65		1	TAG		
*lrRNA*	N	12,656–13,983	1328					
*tRNA^Val^*	N	13,984–14,055	72			TAC		
*srRNA*	N	14,056–14,839	784					
A + T rich		14,840–15,831	992	66				
*tRNA^Ile^*	J	14,929–14,994	66			GAT		

*Chrysomya phaonis* not only could cause harm to human health as medical vectors (Norris 1965; Zumpt 1965; Greenberg 1971; Kuhlhorn 1983; Ghandour 1988), but also are significant in forensic science (Harvey et al. 2008). DNA typing of forensic insect specimens offers a quick and reliable alternative, so more and more researchers have started using the whole sequences of mitochondrial genome or part of the genes to identify species of Blowflies (Wells & Williams 2007; Harvey et al. 2008; Desmyter & Gosselin 2009; DeBry et al. 2013).

Here, we provide the entire genome of mitochondrial DNA of *C. phaonis.* The phylogenetic analysis of *C. phaonis* was performed by comparison with other 39 Diptera species mitochondrial genomes (Figure 1). Phylogenetic tree was generated by a neighbour-joining analysis of MEGA 6.0 program (Tamura et al. 2013) using 1000 bootstrap replicates. The N-J tree revealed that *C. phaonis* was placed mostly close to *C. pinguis,* which could not be distinguished with the common adopted COI DNA barcode sequences. So, it may provide some help for the molecular identification of *C. phaonis*, in particular to distinguish *C. phaonis* from its closely related species *C. pinguis*.

**Figure 1.**
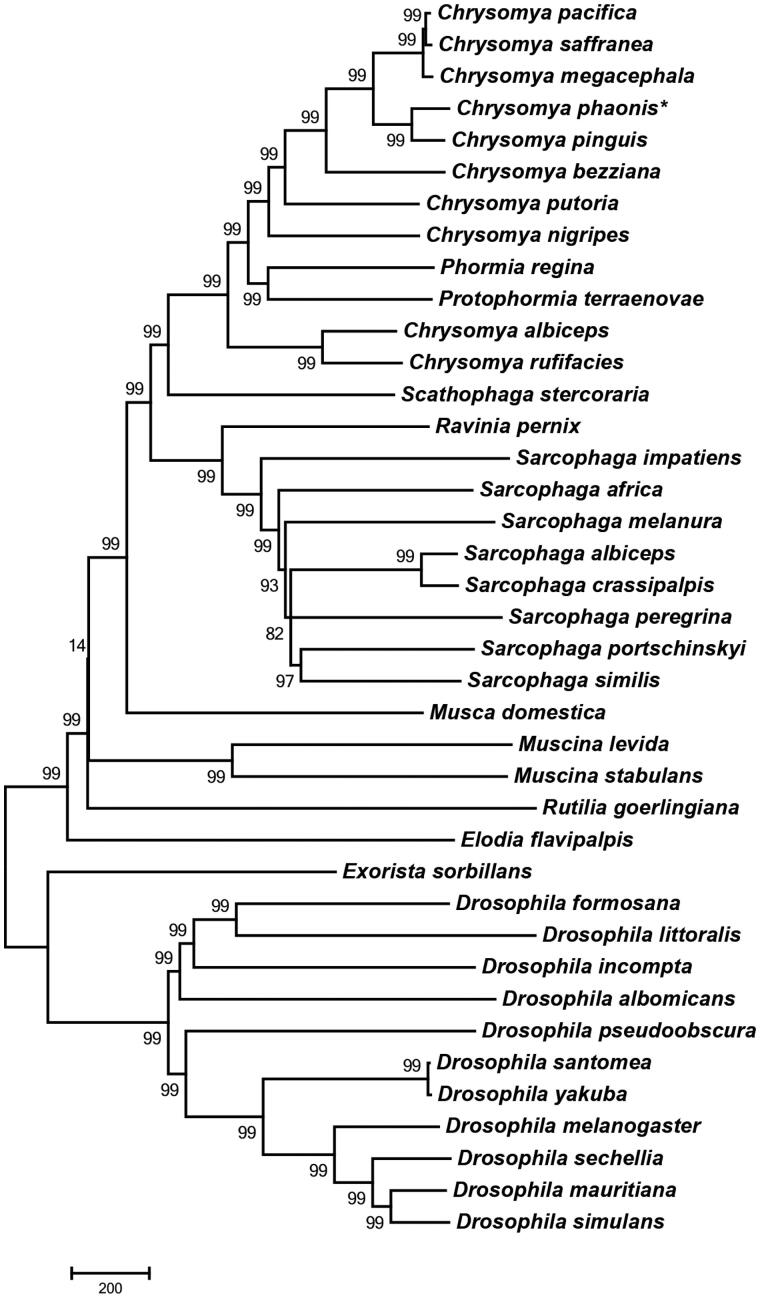
The neighbour-joining (NJ) tree of *C. phaonis* with other 39 Diptera species based on mitochondrial genomes. For each node, the bootstrap support was calculated using 1000 replicates. GenBank accession numbers of mitochondrial genomes used in this hylogeny analysis were listed: *C. albiceps* (NC_019631.1); *C. bezziana* (JX913737.1); *C. megacephala* (KT272775.1); *C. nigripes* (KT444441.1); *C. pacifica* (KP861632.1); *C. pinguis* (KM244730.1); *C. putoria* (AF352790.1); *C. saffranea* (JX913742.1); *C. saffranea* (JX913742.1); *Protophormia terraenovae* (JX913743.1); *Sarcophaga africa* (KM881633.1); *S. albiceps* (NC_028413.1); *S. crassipalpis* (KP861920.1); *S. impatiens* (NC_017605.1); *S. melanura* (NC_026112.1); *S. peregrina* (NC_023532.1); *S. portschinskyi* (NC_025574.1); *S. similis* (NC_025573.1); *Scathophaga stercoraria* (KM200724.1); *Musca domestica* (KT444442.1); *Muscina stabulans* (NC_029487.1); *M. stabulans* (NC_026292.1); *Elodia flavipalpis* (NC_018118.1); *Exorista sorbillans* (HQ322500.1); *Ravinia pernix* (NC_026196.1); *Rutilia goerlingiana* (NC_019640.1); *Phormia regina* (KC005712.1); *Drosophila albomicans* (NC_027937.1); *Drosophila formosana* (NC_028518.1); *D. incompta* (NC_025936.1); *D. littoralis* (FJ447340.1); *D. mauritiana* (AF200831.1); *D. mauritiana* (NC_005779.1); *D. melanogaster* (KT174474.1); *D. pseudoobscura* (NC_018348.1); *D. santomea* (KF824869.1); *D. sechellia* (AF200832.1); *D. simulans* (AY518674.1); *D. yakuba* (KF824899.1).
